# Connectomics in Brain Aging and Dementia – The Background and Design of a Study of a Connectome Related to Human Disease

**DOI:** 10.3389/fnagi.2021.669490

**Published:** 2021-10-07

**Authors:** Ann D. Cohen, Ricardo Bruña, Yue-Fang Chang, Yu Cheng, Jack Doman, Ted Huppert, Tae Kim, Fernando Maestu, Rebecca E. Roush, Beth E. Snitz, James T. Becker

**Affiliations:** ^1^Department of Psychiatry, The University of Pittsburgh, Pittsburgh, PA, United States; ^2^Department of Experimental Psychology, Universidad Complutense de Madrid, Pozuelo de Alarcón, Madrid, Spain; ^3^Department of Neurosurgery, The University of Pittsburgh, Pittsburgh, PA, United States; ^4^Department of Statistics, The University of Pittsburgh, Pittsburgh, PA, United States; ^5^Department of Biostatistics, The University of Pittsburgh, Pittsburgh, PA, United States; ^6^Department of Electrical Engineering, The University of Pittsburgh, Pittsburgh, PA, United States; ^7^Department of Radiology, The University of Pittsburgh, Pittsburgh, PA, United States; ^8^Department of Neurology, The University of Pittsburgh, Pittsburgh, PA, United States; ^9^Department of Psychology, The University of Pittsburgh, Pittsburgh, PA, United States

**Keywords:** aging, MRI, amyloid PET imaging, magnetoencepalography, Connectome Related to Human Disease, neuropsychology

## Abstract

The natural history of Alzheimer’s Disease (AD) includes significant alterations in the human connectome, and this disconnection results in the dementia of AD. The organizing principle of our research project is the idea that the expression of cognitive dysfunction in the elderly is the result of two independent processes — the neuropathology associated with AD, and second the neuropathological changes of cerebrovascular disease. Synaptic loss, senile plaques, and neurofibrillary tangles are the functional and diagnostic hallmarks of AD, but it is the structural changes as a consequence of vascular disease that reduce brain reserve and compensation, resulting in an earlier expression of the clinical dementia syndrome. This work is being completed under the auspices of the Human Connectome Project (HCP). We have achieved an equal representation of Black individuals (vs. White individuals) and enrolled 60% Women. Each of the participants contributes demographic, behavioral and laboratory data. We acquire data relative to vascular risk, and the participants also undergo *in vivo* amyloid imaging, and magnetoencephalography (MEG). All of the data are publicly available under the HCP guidelines using the Connectome Coordinating Facility and the NIMH Data Archive. Locally, we use these data to address specific questions related to structure, function, AD, aging and vascular disease in multi-modality studies leveraging the differential advantages of magnetic resonance imaging (MRI), functional magnetic resonance imaging (fMRI), MEG, and *in vivo* beta amyloid imaging.

## Introduction

The natural history of Alzheimer’s Disease (AD) includes significant alterations in the human connectome, and this disconnection results in the dementia of the Alzheimer’s type (DAT). Data from structural and functional magnetic resonance imaging (MRI) (Dai and He, 2014; Prescott et al., 2014), as well as magnetoencephalopathy (MEG) (Lopez-Sanz et al., 2019) and electroencephalography (Maestu et al., 2019; Babiloni et al., 2020) all demonstrate significant changes in neural networks even prior to the onset of clinical dementia. While such changes are not explicit in the popular A/T/N (amyloid/tau/neurodegeneration) model of AD (Jack et al., 2016), they appear to be an early consequence of the accumulation of beta amyloid (Busche and Konnerth, 2016; Nakamura et al., 2017), and thus may be an early warning sign of impending neurodegeneration. Indeed, models of the natural history of AD that propose that the loss of synapses is one of the first pathological stages of AD (Selkoe, 2002), imply changes in the connectome.

In 2016 the University of Pittsburgh was awarded funds by the National Institute on Aging under the Connectomes Related to Human Disease^1^ of the Human Connectome Project.^2^ Our project is organized around the idea that the natural history of AD is affected by multiple independent factors (Ewers et al., 2011), and that the expression of cognitive dysfunction is the result of independent processes including AD and vascular-related neuropathology. Here we describe the general organization of the *Connectomics in Brain Aging and Dementia* project, the sampling frame, a brain imaging protocols, and the behavioral/cognitive data that were acquired as part of the study. All of the study data are currently being uploaded to the *Connectome Coordination Facility*^3^ and the NIMH National Data Archive.^4^

To accomplish the study goals, we acquired neuropsychological data, as well as brain structural and functional (functional MRI, MEG) imaging, and positron emission tomography (PET) imaging of *in vivo* of brain amyloid with Pittsburgh Compound B (PET-PiB). We used different measures of brain function because fMRI and MEG rely on fundamentally different biological processes to generate “signal” (Tsvetanov et al., 2015), and this has the potential to provide critical information about the uncoupling of the neural and vascular components in AD (and possibly in normal aging) (Zlokovic, 2011). Because the MEG signal is derived from post-synaptic currents, and fMRI signal also includes a vascular response, they may expose different sources of the disconnection (i.e., degeneration vs. vascular). We also acquire a direct measure of cerebrovascular function – an MRI-based measure of cerebral blood flow, as well as a direct measure of AD pathology using *in vivo* amyloid imaging. These data provide the opportunity to examine the relationship between amyloid deposition and local and distant connectivity (Zhou et al., 2015) among individuals with and without cognitive impairment.

## Methods

### Study Design

This is a longitudinal, community-based study of brain structural and functional connectivity among cognitively normal and cognitively impaired individuals aged 50–89 years.

### Recruitment Sources

There are currently two primary portals of entry into the study: the University of Pittsburgh Alzheimer’s Disease Research Center^5^ and the Pitt + Me web portal (primarily to recruit Black individuals and Whites without college education).^6^ Additional individuals were identified through active links with the Heart SCORE Study (Bambs et al., 2011), the Long Life Family Study (Newman et al., 2011), and by word of mouth.

### Study Protocol

All study participants are tested/scanned over three days. On Day One, all study enrollees complete the informed consent process and the intake forms. They are then escorted to the MR Research Center (MRRC) and where they complete the two fMRI tasks (motor, working memory), and the structural imaging. Following a break, the individuals complete the behavioral tests that are *not* components of the NIH Toolbox. On Day Two, the participants undergo a brief exam and fasting blood tests. They are then taken back to the MRRC where they undergo diffusion imaging, task free fMRI and the language/math task fMRI; they then complete all the NIH Toolbox tests. On Day Three the participants undergo MEG and PET-PiB scanning; this is scheduled approximately 1 week after the last MRI scanning session (to avoid any interference of the MRI on the MEG data). The participants are escorted to the Center for Advanced Brain Magnetic Source Imaging^7^ where they are prepared for the MEG scan, and complete task training. Once in the magnetically shielded room, the individuals complete task free MEG, and one task MEG (working memory). Individuals will then take for a short break and for the placement of the electrodes for the motor stimulation; then they will complete the Language/Math and Motor MEG task scans. Following a break for either a snack or lunch, the participants are escorted to the UPMC PET Facility for their PiB scan.

#### Diagnostic Evaluation

Each participant undergoes a brief neuropsychological test battery for group classification purposes. The test battery is based on that of the ADRC and includes the Montreal Cognitive Assessment (MoCA) (Nasreddine et al., 2005), verbal fluency, a 30-item visual naming test (Saxton et al., 2000), Trailmaking (Reitan, 1958; Reitan and Wolfson, 1994), verbal free recall (Welsh et al., 1991, 1994), and the Rey-Osterreith Complex Figure (Rey, 1941). Classification decisions were made independently by JTB, and BES and any differences were resolved in a group discussion. We use the ADRC classification scheme (Lopez et al., 2000) for AD, MCI (both amnestic and non-amnestic), and Subjective Cognitive Complaints (SCC).

#### Neuropsychological Tests and Questionnaires

The individual tests and questionnaires that serve as outcome variables include items from the *NIH Toolbox*,^8^ the *Promis* battery,^9^ and additional paper-and-pencil tests (see Supplementary Tables 1–3). The questionnaires cover symptomatology, personality, diet, and exercise.

#### Brain Imaging

##### Magnetic Resonance Imaging Scanning

We use Siemens Prisma 3-Tesla 64-channel systems equipped with Connectome level gradients operating at 80mT/m. They are equipped with fMRI presentation systems including E-Prime, a MR compatible video projector, and Celeritas response gloves.

The MRI scanning is completed in two 90-min sessions over two days. The scan sequences include: T1-weighted MP-RAGE, T2-weighted SPACE image, FLAIR, susceptibility weighted imaging, diffusion tensor imaging, task-free functional MRI, task-based fMRI, and arterial spin labeling (see Supplementary Table 4).

The tasks used were those described for the HCP and, with one exception, used the stimuli provided by the HCP; the exception was the N-back task. For that task all of the original photographs of faces were of White individuals; we substituted photos of Black individuals so that half of all of the N-back trials used White faces, and half Black faces. The same race was used for all of the stimuli within a trial (i.e., race could not be used to select responses).

All the MRI data are processed locally through the HCP pipeline, as modified to work in the local environment. The raw data are stored on an XNAT server^10^ and pushed to a receiving server at Washington University in St. Louis for processing by the Connectome Coordination Facility and eventual upload to the on-line, public HCP database.

##### Magnetoencephalopathy Recording

Magnetoencephalography (MEG) studies are completed on an Elekta-Neuromag Vectorview 306 MEG system. The whole-scalp neuromagnetic measurement system uses 102 triple sensors – 102 magnetometers and 204 planar gradiometers – in a helmet-shaped array. The locations of three cardinal anatomical landmarks (nasion, and two preauricular points) and of four head localization coils are digitized prior to each MEG study using a 3D-digitizer (ISOTRAK; Polhemus, Inc., Colchester VT) to define the subject-specific Cartesian head coordinate system. 30–50 anatomical points are digitized on the head surface to provide for more accurate co-registration of the MEG data with the reconstructed volumetric MR image. Eye movements are measured and recorded simultaneously with the MEG. The MEG sensor unit, the floor-mounted gantry, the subject chair and bed, together with the patient audio-visual monitoring and stimulus delivery systems are contained in a magnetically shielded room.

Once a subject is comfortably positioned in the MEG machine, a short electrical signal is sent to the head coils enabling their localization with respect to the MEG sensor array. The MEG data are acquired at a sampling rate of 1 kHz, with on-line filtering of 0.10–330 Hz. The acquisition includes two memory tasks, as well as 10 min of “resting state” data – 5 min with eyes open followed by 5 min with eyes closed. At the end of the scan, we collect 2 min of “empty room” data to assess the validity of any signal in the test conditions.

Recordings were filtered offline using a tempo-spatial filtering algorithm (tSSS, correlation window 0.9, time window 10 s) (Taulu and Simola, 2006) to eliminate magnetic noise originating outside the head and to compensate for head movements.

The raw data are stored on an XNAT server and are pushed to the NDA for eventual inclusion in the study database (C3159).

##### Positron Emission Tomography Amyloid Imaging

The PET amyloid tracer, Pittsburgh Compound B (PiB) is synthesized by a simplified radiosynthetic method based on the captive solvent method (Wilson et al., 2000; Price et al., 2005). High specific activity (> 0.50 Ci/μmol at time of injection) PiB (15 mCi) is injected over 20 s and the participant then relaxes quietly in a chair for ∼25 min, after which they are positioned in the scanner. A windowed transmission scan (10 min) is acquired for attenuation correction, followed by a 30 min PiB PET study (6 × 300 s frames).

The Siemens/CTI ECAT HR + scanner gantry is equipped with a Neuro-insert (CTI PET Systems) to reduce the contribution of scattered photon events (Weinhard, 1998). Positron emission tomography data are reconstructed using filtered back-projection (Fourier rebinning and 2D backprojection with Hann filter: kernel FWHM = 3 mm). Data are corrected for photon attenuation, scatter (Watson et al., 1997), and radioactive decay. The final reconstructed PET image resolution is ∼ 6 mm (transverse and axial) based on in-house point source measurements.

The raw data are stored on an XNAT server and are pushed to the NDA for inclusion in the study database (C3159). The data include the dynamic images as well as a single SUV image.

#### Imaging Data Processing (Local)

All the MRI data are pushed to the HCP CCF XNAT server where they are processed using standard quality control measures, and analysis via the HCP Pipeline. The processed data are made available by the CCF. The MEG and PET data are saved to the NIMH Data Archive as.FIF files (MEG) and DICOM images (PET SUV images). What follows below is the description of the local processing of these data.

##### Magnetic Resonance Imaging Structural Image Processing

We briefly describe here the HCP Minimal Processing Pipelines that are implemented at the CCF prior to the release of the data [See Glasser, et alia (Glasser et al., 2013) for details]. There are three main components to the structural data processing. In the first steps, the goal is to produce a “native” structural space for each subject, align the T1 and T2 images, perform a bias field correction, and co-register the structural volumes into MNI space. The second component which uses FreeSurfer extensively, segments these volumes into predefined subcortical and cortical regions. It also reconstructs cortical surfaces and performs the standard surface registration to the FreeSurfer atlas. Finally, in the third step all the NIFTI and GIFTI surface files are created that can then be used in the Connectome Workbench.

In addition, we also process all the MP-RAGE data through Computational Anatomy Toolbox (CAT12) for SPM.^11^ This process provides the basis for a range of morphological analysis methods, including voxel-based morphometry, surface-based morphometry, deformation-based morphometry, and region- or label-based morphometry.

##### Positron Emission Tomography Processing

The PET data are processed using PMOD^12^ and Freesurfer software packages. Correction for subject motion during the multi-frame PET scan is performed using frame-to-frame registration procedure. The PET data are averaged to generate images that correspond to the 50–70 min post-injection uptake. The anatomical T1-weighted MR image is reoriented along the anterior-posterior commissure and the averaged PET images are co-registered to the reoriented MR image. Freesurfer software is used for MR bias field correction, automated ROI parcellation and tissue segmentation. The Freesurfer ROI parcellations are converted into an ROI template and ROI sampling of the PET images is performed to include anterior cingulate, frontal cortex, parietal, precuneus, lateral temporal cortex, primary visual cortex, hippocampus, anterior ventral striatum, thalamus, pons, and cerebellum.

Regional standardized uptake value (SUV) measures are computed for PiB by normalizing tissue uptake to the injected radioligand dose and body mass. Each regional SUV is normalized to a reference ROI in the cerebellum to generate the SUV ratio (SUVR). Cortical SUVRs were measured in anterior cingulate cortex, the superior frontal cortex, orbital frontal cortex, lateral temporal cortex, parietal lobe, precuneus, and the anterior ventral striatum regions and averaged across hemispheres. The volume-weighted average of these seven SUVR values constituted the Global SUVR. The SUVR in each area is compared to a region-specific cut-off determined by sparse k-means clustering; those scores above the cut-off are considered “positive”. If *any* of the regions was considered “PiB Positive,” then the Global rating was set to positive (Cohen et al., 2013).

##### Magnetoencephalography Signal Processing

Ocular, muscular and jump artifacts are identified using an automatic procedure from the Fieldtrip package (Oostenveld et al., 2011). The remaining data are segmented into 4 s epochs of artifact-free activity using only the magnetometer data (Garces et al., 2017). An ICA-based procedure is used to remove the electrocardiographic component.

###### Source Reconstruction

Artifact-free epochs are filtered between 2 and 40 Hz, to remove both low frequency noise and network line artifact. The epochs are padded with 2 s of real signal from both sides prior to the filtering to prevent edge effects inside the data. The source model consists of 2459 sources placed in a homogeneous grid of 1 cm in MNI template, then linearly transformed to subject space by warping the subject T1-weighted MRI into the MNI template. The lead field is calculated using a single shell (the brain-skull interface) generated from the T1 MRI using Fieldtrip^13^ and a modified spherical solution (Nolte, 2003). A Linearly Constrained Minimum Variance beamformer (Van Veen et al., 1997) is used to obtain the source time series by using the computed lead field and building the beamforming filter with the epoch-averaged covariance matrix and a regularization factor of 5% of the average channel power.

###### Spectral Analysis

The estimated spatial filters are used to reconstruct the source-space time series for each epoch and source location. MEG power spectra are calculated between 2 and 40 Hz for every clean epoch using a Hann taper, with 0.25 Hz steps. The resulting spectra for each trial are averaged to build the final spectrum for each source. The obtained power is normalized with the overall power in Rey (1941), Reitan (1958), Welsh et al. (1991, 1994), Reitan and Wolfson (1994), Van Veen et al. (1997), Watson et al. (1997), Weinhard (1998), Lopez et al. (2000), Saxton et al. (2000), Wilson et al. (2000), Selkoe (2002), Nolte (2003), Nasreddine et al. (2005), Price et al. (2005), Rosano et al. (2005), Schinka et al. (2005), Taulu and Simola (2006), Erickson et al. (2010, 2013), Bambs et al. (2011), Ewers et al. (2011), Newman et al. (2011), Oostenveld et al. (2011), Zlokovic (2011), Cohen et al. (2013), Glasser et al. (2013), Lambert et al. (2013), Prescott et al. (2014), Hughes et al. (2015), Tsvetanov et al. (2015), Zhou et al. (2015), Busche and Konnerth (2016), Jack et al. (2016), Garces et al. (2017), Nakamura et al. (2017), Lopez-Sanz et al. (2019), Maestu et al. (2019), and Babiloni et al. (2020) Hz. The normalized spectra of all the sources in each brain lobe were averaged, obtaining one value per frequency step, brain lobe and subject. Last, we calculated the relative power per lobe in each of the standard frequency bands: Delta (2–4 HZ), Theta (4–8 Hz), Alpha (8–12 Hz), Beta (12–30 Hz), and Gamma (30–40 Hz).

##### Genotyping

We are genotyping each study participant for 21 previously identified susceptibility genes (Lambert et al., 2013) including APOE^∗^4 (see Supplementary Table 5). The genetic information is also uploaded to the NDA but requires special permissions for access.

##### Measures Related to Risk/Protection From Cognitive Impairment

Each of the study subjects provides additional data related to risk for and protection from cognitive impairment based on studies from our prior research. With regard to exercise and motor function, each subject wears an activity monitor (Erickson et al., 2010, 2013) for five days, and we query them about the amount of walking per week, estimate the number of kilocalories burned per week, and measure gait speed (Rosano et al., 2005) (in addition to the motor tasks used by the NIH Toolbox). Each participant completes the Florida Cognitive Activities scale to obtain a measure of activities that might affect cognitive and brain health (Schinka et al., 2005; Hughes et al., 2015).

On Day Two, we measure blood pressure, height, weight, and waist-hip ratio (Mukamal et al., 2003). Laboratory measures include a fasting lipid profile (Wong et al., 2010), cystatin-c, homocysteine (Longstreth et al., 2004), and inflammatory markers (Tracy et al., 1997; Fornage et al., 2008; Braskie et al., 2014).

#### Quality Control/Assurance Procedures

##### Quality Control

###### Magnetic Resonance Imaging Scanner

The MRRC has QC/QA procedures and American College of Radiology certification in place for all scanners. These include daily signal stability scans for echo planar imaging (1% maximum RMS over a continuous 30-min acquisition with a 64 × 64 matrix size) and daily signal-to-noise measurements with the standard RF head coil. In addition to the daily QC testing of the MRI scanner, each imaging protocol is examined visually prior to submitting it to the local data archive. The scans are checked immediately by a member of the Imaging Team and repeated if necessary.

###### Positron Emission Tomography Scanner

QC/QA procedures are run according to the University of Pittsburgh PET Facility Standard Operating Procedures HR + Quality Assurance Task Schedule. The “Daily QC” protocol runs a scan that is compared to the last standard that was written into the database. That is, the standard that was written by the *Norm 2D and ECF (Customer)* protocol. The resulting deviation between scans must be less than 2.5. The protocol uses the internal rod sources of the gantry, so no phantoms are used.

###### Magnetoencephalography Scanner

The operating status of the *Elekta NeuroMag* system is tested daily. This includes determining that there is a sufficient level of liquid helium, calibrating and tuning the sensors, determining the proper functioning of the magnetic shielding producing a sufficiently low ambient magnetic interference level.

###### Neuropsychological Testing

Clinical Team Leader Dr. Snitz trains the staff who are responsible for administering and/or scoring questionnaires or paper-and-pencil tests as she does within the ADRC.

##### Quality Assurance

###### Magnetic Resonance Imaging Scanner

We use the ADNI phantom as a reference tool for our structural and functional images.

###### Positron Emission Tomography Scanner

The ^68^Ge phantom is run at on a weekly basis to check for changes in the scanner calibration or changes in uniformity. Four times each year the following procedures are performed in order: Full ASIC Bucket Setup; System Normalization; Daily QC; and, Scanner/Well Counter Cross Calibration.

###### Magnetoencephalography Scanner

Prior to and after every scan we record 2 min of empty room data to measure ambient magnetic noise. We complete a simple spectral analysis and then save the raw data and spectra. This allows for monitoring the noise level and system status over time to help identify changes in the background environment.

###### Neuropsychological Testing

Dr. Snitz reviews the scoring of all questionnaires and paper-and-pencil tests. Every six months a sample of ten protocols will be “double scored” to ensure inter-rater reliability. Five of these protocols will be repeated annually to check for scoring drift.

## Preliminary Results

The data acquired through this protocol are and will continue to be uploaded to the CCF and NDA. However, the team has completed some initial analyses to help to better explicate the participants who had enrolled in the study by March 31, 2020. The data provides critical information about the relationship between the breakdown in functional and structural connectivity and the expression of cognitive impairment along the AD-pathology continuum. Because of our unique sampling frame, we have data from participants who are less likely to enroll in biomedical research studies, and this has revealed several aspects of the normal/pathological aging spectrum that were previously under-appreciated.

The study was reviewed and approved by the University of Pittsburgh Human Research Protection Office. All participants signed written statements of Informed Consent prior to initiation of any research procedures.

### Subjects

A total of 472 individuals inquired about the study and of these, 208 either chose not to enroll or failed the initial screening questions related to MR compatibility (e.g., metal implants) or medical history (e.g., clinical stroke). Twenty-seven individuals were excluded after having signed an informed consent form; as of 31 March 2020, 227 individuals had enrolled in the study.

Of these participants, 13 had been diagnosed with DAT; these individuals are not described in this report. Sixty-seven study participants (31%) entered via the ADRC; 97 (45%) came through Pitt + Me, and 27 (13%) were volunteers from the community. Twenty-one participants (10%) entered through HeartScore or the LLFS.

We compared the characteristics of the participants initially classified as having normal cognition to those with some degree of impairment. There were two subgroups among the Cognitively Normal participants: those who reported no limitations in their cognition and those who reported significant concerns [Subjective Cognitive Complaints (SCC)]. There were also two subgroups among the cognitively impaired participants: those who reported no concerns or loss of abilities [Impaired Without Complaints (IWOC)], and those who reported loss of abilities (i.e., MCI) (see Tables 1–3).

**TABLE 1:** Characteristics of study participants as a function of initial classification.

	Study groups		Effect size^1^
	Normal cognition	Impaired cognition	
Number	121	93	
Age	65.6 (8.0)	62.8 (9.7)	0.03*
Education	16.0 (3.1)	13.9 (2.8)	0.13*
Sex [Percent (N) Male]	30.6 (37)	30.4 (32)	0.04
Race [Percent (N) Caucasian]	59.5 (72)	34.4 (32)	0.29*
Handedness [Percent (N) Right]	95.7 (112)	90.0 (81)	0.11*
APOE*4 Present [Percent(N)]	26.1 (29)	34.9 (30)	0.10
Montreal Cognitive Assessment	26.5 (2.3)	23.4 (2.7)	0.44*
Wide Range Achievement Test – 4	63.8 (5.1)	59.1 (7.8)	0.18*
Walk Endurance – *2min distance*	544.4 (177.2)	502.1 (87.0)	0.02
Gait Speed – *4meter walk* – *time*	3.4 (0.5)	3.4 (0.7)	0.001
Oral reading	6.9 (3.6)	4.6 (2.8_	0.12*
DCCS	29.3 (1.0)	28.2 (2.0)	0.13*
Flanker Inhibitory Control	19.9 (0.8)	19.9 (0.6)	0.00
Pattern Comparison	41.7 (6.3)	38.5 (7.6)	0.05*
Picture Sequence Memory	11.7 (6.8)	7.3 (4.7)	0.14*
Crystalized Cognition	113.5 (9.3)	105.0 (9.1)	0.21
Fluid Cognition	97.5 (9.1)	88.4 (9.2)	0.25*
Total Cognition	105.8 (8.8)	95.4 (7.4)	0.40*
Promis Abilities	30.8 (7.2)	28.1 (8.0)	0.03*
Promis Concerns	16.1 (7.3)	17.0 (7.5)	0.004
Life Satisfaction	19.9 (7.4)	20.1 (10.7)	0.000
Meaning	29.2 (15.3)	30.1 (14.7)	0.001
Positive Affect	18.8 (13.1)	21.2 (15.6)	0.01
Sadness	8.9 (2.4)	9.2 (2.2)	0.004
Self-Efficacy	19.6 (9.4)	18.5 (9.7)	0.003

*^1^Cramer’s V for categorical data; Cohen’s f^2^ for continuous data. ^∗^*p* < 0.05.*

**TABLE 2:** Characteristics of cognitively normal participants by subgroup.

	Study groups		Effect size^1^
	Healthy controls	Subjective complaints/No impairments	
Number	104	17	
Age	65.3 (8.2)	68.6 (6.9)	0.02
Education	16.2 (2.9)	16.4 (4.7)	0.001
Sex [Percent (N) Male]	26.9 (28)	52.9 (9)	0.20*
Race [Percent (N) Caucasian]	56.7 (59)	76.5 (13)	0.15
Handedness [Percent(N) Right]	96.1 (99)	92.9 (13)	0.05
APOE*4 Present [Percent(N)]	27.2 (25)	30.8 (4)	0.01
Montreal Cognitive Assessment	26.4 (2.4)	26.9 (1.4)	0.008
Wide Range Achievement Test – 4	64.1 (5.0)	57.5 (2.1)	0.095
Walk Endurance – *2min distance*	523.0 (82.7)	677.5 (419.0)	0.10*
Gait Speed – *4meter walk* – *time*	3.4 (0.5)	3.3 (0.4)	0.003
Oral Reading Recognition	6.9 (3.8)	6.7 (2.4)	0.000
Dimensional Change Card Sort Test	29.4 (0.8)	28.7 (1.4)	0.07*
Flanker Inhibitory Control	19.9 (0.8)	20.0 (0.0)	0.002
Pattern Comparison	41.7 (6.1)	41.4 (7.6)	0.000
Picture Sequence Memory	11.8 (6.8)	11.2 (6.9)	0.001
Crystalized Cognition	113.3 (9.6)	114.4 (7.8)	0.002
Fluid Cognition	97.6 (8.9)	97.0 (9.9)	0.001
Total Cognition	105.8 (8.8)	106.0 (9.0)	0.000
Promis Abilities	31.5 (7.0)	27.3 (7.5)	0.04*
Promis Concerns	15.4 (7.0)	20.4 (8.1)	0.06*
General Life Satisfaction	20.0 (7.8)	19.1 (4.5)	0.002
Meaning and Purpose	30.4 (15.9)	21.9 (8.5)	0.04*
Positive Affect	19.3 (13.3)	15.8 (11.7)	0.009
Sadness	8.8 (2.4)	9.0 (2.5)	0.001
Self-Efficacy	20.0 (9.5)	17.2 (8.6)	0.011

*^1^Cramer’s V or Cohen’s f^2^^∗^*p* < 0.05.*

**TABLE 3:** Characteristics of cognitively impaired participants by subgroup.

	Study groups		Effect size^1^
	Mild cognitive impairment	Impaired/No complaints	
Number	52	41	
Age	64.9 (10.4)	60.5 (8.0)	0.06*
Education	14.5 (2.7)	13.1 (2.7)	0.07*
Sex [Percent (N) Male]	28.9 (15)	41.5 (17)	0.13
Race [Percent (N) Caucasian]	48.1 (25)	17.1 (7)	0.32*
Handedness [Percent(N) Right]	92.0 (46)	87.5 (35)	0.08
APOE*4 Present [Percent(N)]	31.3 (15)	39.5 (15)	0.09
Montreal Cognitive Assessment	23.3 (2.6)	23.5 (3.0)	0.001
Wide Range Achievement Test – 4	60.9 (6.8)	56.1 (8.5)	0.10
Walk Endurance – *2min distance*	481.9 (86.3)	528.2 (82.1)	0.08*
Gait Speed – *4meter walk* – *time*	3.6 (0.7)	3.3 (0.6)	0.04
Oral Reading Recognition	4.9 (2.6)	4.1 (2.9)	0.02
Dimensional Change Card Sort Test	28.2 (2.0)	28.1 (2.1)	0.002
Flanker Inhibitory Control	19.9 (0.8)	20.0 (0.2)	0.01
Pattern Comparison	36.6 (8.3)	40.6 (6.3)	0.08
Picture Sequence Memory	7.1 (4.4)	7.6 (5.0)	0.003
Crystalized Cognition	106.6 (9.0)	103.2 (9.1)	0.04
Fluid Cognition	86.5 (9.2)	90.3 (8.9)	0.05
Total Cognition	95.5 (7.8)	95.3 (7.0)	0.000
Promis Abilities	24.7 (7.9)	32.6 (5.7)	0.32*
Promis Concerns	20.3 (7.5)	12.7 (4.6)	0.35*
General Life Satisfaction	19.6 (8.7)	20.7 (12.6)	0.003
Meaning and Purpose	26.5 (13.2)	34.1 (15.1)	0.07*
Positive Affect	18.1 (12.3)	24.7 (18.1)	0.05
Sadness	8.8 (2.1)	9.5 (2.4)	0.03
Self-Efficacy	17.0 (8.0)	20.2 (11.2)	0.03

^1^Cramer’s V or Cohen’s f^2^. *p < 0.05.

The proportion of Black individuals was greater within the cognitively impaired group, as was the proportion reporting being left-handed. As would be expected, the Crystallized and Fluid Intelligence measures from the NIH Toolbox were significantly lower among the impaired participants.

The two subgroups of individuals who were cognitively normal did not differ in terms of age, years of education, distribution of men and women, race, or handedness (see Table 2). The MoCA scores were equivalent, but the individuals in the SCC group performed more poorly on the Wide Range Achievement Test. The SCC group reported more cognitive concerns, and lower scores on the measure of Meaning and Purpose. The latter indicates more hopelessness, less goal-directedness, less optimism, and weaker feelings that their life is “worthy”.^14^

Between the two subgroups of individuals with Impaired Cognition those in the IWOC group were younger, less well educated, and more likely to self-identify as Black; they had decreased physical endurance (see Table 3). The IWOC group reported significantly *better* cognitive abilities (higher scores) and *fewer* cognitive concerns (lower scores) than the people in the MCI group. They reported higher scores on the Meaning and Purpose questions from the Promis battery.

The participants with MCI had significantly lower scores on the Promis Cognitive Abilities questionnaire relative to the healthy controls [*t*(132) = −4.39, *d* = 0.76], and reported significantly more concerns about their cognition [*t*(132) = 3.77, *d* = 0.66]. By contrast, the individuals in the IWOC group did not differ significantly on the Cognitive Abilities scale [*t*(125) = 1.20, *d* = 0.21], and reported *fewer* concerns about their cognition than did the healthy controls [*t*(125) = −2.54, *d* = 0.45]. Finally, when we compared the MCI and IWOC groups, we found that those with MCI had lower scores on the Cognitive Abilities questionnaire [*t*(75) = −5.00, *d* = 1.15], and reported significantly more concerns [*t*(75) = 6.12, *d* = 1.41] about their cognition than the IWOC group.

### Structural Magnetic Resonance Imaging Data

We calculated an index of the cortical thickness of critical temporal lobe areas including the fusiform gyrus, entorhinal cortex, and the inferior and middle temporal gyri (Jack et al., 2017) using values taken from the standard output of the HCP pipeline. We then classified each case as “normal” or “atrophic” based on the standard cut-off of + 2.70 mm (see Table 4).

**TABLE 4:** Summary neuroimaging findings among four subject groups.

	Groups				Effect size ^1^
	Healthy controls	Subjective complaints	Impaired without complaints	Mild cognitive impairment	
Number	96	17	39	46	
Temporal Lobe Cortical Thickness ^2^	2.80 (0.24)	2.72 (0.37)	2.72 (0.27)	2.68 (0.28)	0.04
Grey Matter Atrophy [Percent (N)] ^3^	21.9 (21)	29.5 (5)	25.6 (10)	37.0 (17)	0.14
PiB SUVR^4,5,6^					
Ant. Cing.	1.29 (0.33)	1.45 (0.11)	1.12 (0.44)	1.29(0.36)	0.07*
Sup. Front.	1.22 (0.30)	1.36 (0.08)	1.08 (0.36)	1.22 (0.35)	0.07*
Orb. Front.	1.27 (0.30)	1.40 (0.09)	1.11 (0.43)	1.26 (0.36)	0.07*
Lat Temp.	1.18 (0.24)	1.31 (0.07)	1.06 (0.29)	1.18 (0.26)	0.08*
Parietal	1.24 (0.25)	1.35 (0.08)	1.09 (0.33)	1.22 (0.28)	0.08*
Precuneus	1.32 (0.34)	1.46 (0.09)	1.13 (0.42)	1.30 (0.37)	0.08*
Ant. Vent. Striatum	1.29 (0.20)	1.43 (0.12)	1.14 (0.33)	1.28 (0.31)	0.11*
PiB Positive [Percent (N)]	32.5 (26)	40.0 (6)	5.7 (2)	22.2 (10)	0.25*

*^1^Cohen’s f^2^ for continuous variables; Cramer’s *V* for categorical data.*

*^2^Average of the cortical thickness values from the fusiform gyrus, entorhinal cortex, and the middle and inferior temporal lobe.*

*^3^Abnormal thickness < 2.70 mm (Jack et al., 2017).*

*^4^ANC – Anterior Cingulate Cortex; FRC – Frontal Cortex; LTC – Lateral Temporal Cortex; PAR – Parietal Cortex; PRC – Precuneus; AVS – Anterior Ventral Striatum.*

*^5^PiB SUVR Cut-off scores: Anterior Cingulate = 1.469; Anterior Ventral Striatum = 1.372; Superior Frontal = 1.333; Orbitofrontal = 1.387; Insula = 1.296; Lateral Temporal = 1.278; Parietal = 1.344; Posterior Cingulate = 1.495; Precuneus = 1.508; Global = 1.346.*

^6^Participants with PiB data: HC = 80, SCC = 15, IWOC = 35, MCI = 45. *p < 0.05.

The mean cortical thickness differed as a function of group (One-Way Analysis of Variance) [*F*(3,182) = 3.18, *f*^2^ = 0.05, *p* < 0.05]. Furthermore, the rate of abnormal thickness differed significantly between groups (*X*^2^ = 7.87, *df* = 3, *V* = 0.21, *p* < 0.05) with the controls and the IWOC having the lowest rates, and the SCC and MCI groups having the highest.

### Positron Emission Tomography Pittsburgh Compound B Data

Positron emission tomography (PET) data were available from 176 of the individuals enrolled in the study. Table 4 shows the data including the mean SUVR for each of the brain regions used for determining amyloid deposition, as well as the global rate of PiB positivity. There is a significant Main Effect of group (One-Way ANOVA) for each of the seven regions of interest (summed across each hemisphere). In addition, the rate of PiB positivity was significantly different across all groups (chi-square test). However, these effects were due to the lower-than-normal SUVRs in each of the six brain regions for the 35 individuals in the IWOC group compared to the healthy controls (all *d*s > 0.61) and their low rate of PiB positivity (Odds Ratio = 14.0, 95% CI = 1.8−110, Exact Test *p* = 0.002) compared to the controls. Among the normal controls the rate of positivity was greater among the White (51.4%) relative to the Black participants (4.5%; OR = 32.2, 95% CI = 2.7−184; Exact Test *p* = 0.0003).

### Amyloid/Neurodegeneration Classification

We compared the rates of PiB retention and temporal lobe atrophy as a function of the clinical classification (see Table 5). There was a significant difference in the rates of biomarker abnormality across groups (*χ*^2^ = 21.5, *df* = 9, *V* = 0.21, *p* < 0.05). Fifty-eight percent of the normal controls were biomarker negative, which is similar to the rates for the SCC (53%) and MCI (49%) groups. By contrast, the IWOC group was 74% biomarker negative. Among the participants with MCI, 29% had *only* temporal lobe atrophy, while 9.8% had *only* PiB + imaging.

**TABLE 5:** Summary amyloid and atrophy findings among four subject [Percent(N) Within Group].

	Groups			
	Healthy controls	Subjective complaints	Impaired without complaints	Mild cognitive impairment
Number	74	15	34	41
No Abnormality	58.1 (43)	53.3 (8)	73.5 (25)	48.8 (20)
Amyloid Only	21.6 (16)	13.3 (2)	2.9 (1)	9.8 (4)
Atrophy Only	9.5 (7)	6.7 (1)	20.6 (7)	29.3 (12)
Amyloid and Atrophy	10.8 (8)	26.7 (4)	2.9 (1)	12.2 (5)

*Cramer’s V = 0.21, p < 0.05.*

### Magnetoencephalopathy Summary Data

**FIGURE 1:**
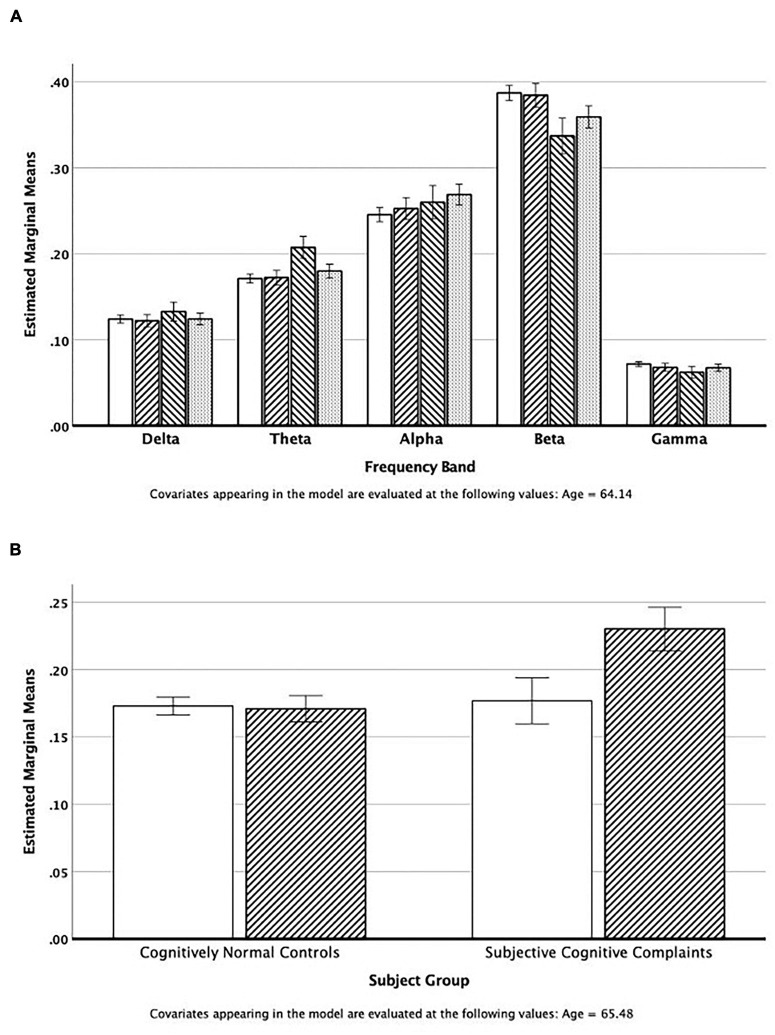
**(A)** Estimated mean power in the five cortical regions adjusted for mean age of the group. The bars represent values for the Normal Controls, IWOC, SCC, and MCI groups, respectively. **(B)** Estimated theta band power in the temporal lobes adjusted for mean age. The open bars are those individuals classified as PiB−, and the cross-hatched bars are for those individuals who are PiB+.

One hundred and eighty-six individuals contributed MEG data that met all quality control standards. We examined the relative power across all five MEG frequency bands in regions of interest (ROI) extracted using the AAL templates (Tzourio-Mazoyer et al., 2002). The repeated measures (band) Analysis of Covariance (age) of temporal lobe power by subject group revealed that the SCC group had elevated theta power compared to the other study groups (see Figure 1A), and decreased beta power. There was no significant association (chi-square tests) between elevated theta power (> 75%tile of normal controls) and race, sex, and APOE^∗^4 status. However, an ANCOVA of temporal lobe theta power revealed a significant interaction between group (NC vs. SCC) and PiB status (positive vs. negative) [*F*(1,64) = 9.11, ξ^2^ = 0.13]. As can be seen in Figure 1B, theta power in the temporal lobe (adjusted for age) is similar in the normal controls (PiB ±) and the PiB- SCC group; power is elevated only in the PiB + SCC participants.

## Discussion

The purpose of this report is to describe the creation of the Connectomics of Brain Aging and Dementia study.^15^ The MRI brain images are being uploaded to the CCF and the behavioral and cognitive data, PET PiB scan regional SUVRs (and raw SUV images), and the raw data from the MEG are being uploaded to the NDA (ID C3159).

### Study Advantages, Limitations, Possible Pitfalls, and How to Counteract Them

When this project was initially proposed to the NIH, we specified that the sample would consist of 50% women and 50% black participants. We further proposed that the 50:50 splits be maintained in each subject group. While we were able to achieve this goal in our sample of healthy controls, some subgroups of participants did not conform to these expectations which in fact reveals much about the characteristics of those phenotypes. We believe that the single biggest advantage of using data derived from this study, and which will continue to be acquired and deposited for public consumption, is the composition of the study sample. We found that by carefully tailoring our public face on Pitt + Me we were able to recruit individuals across a wide range of socioeconomic strata as well as a high rate of Black volunteers. While many studies successfully enroll Black participants at a rate consistent with the population distribution, we specifically chose to oversample Blacks. The individuals that we ended up enrolling, both White and Black, were frequently new to research, and often had relatively low health-related knowledge. In our view, these are the people who need to be enrolled in studies such as COBRA in order to see the process of aging and neurodegeneration as it exists in the broader community.

However, we learned several things about the execution of the protocol that had not been self-evident prior to the study. First, and perhaps most important, the research participants require a great deal of “hands-on” care than the typical research participant. In the end, each participant is assigned to a Research Associate who is, in effect, a concierge. They escort the participant around the medical center for the various procedures. They may be an examiner or interviewer who sits with the participant during neuropsychological testing or completion of healthcare questionnaires. They may take the participant to the cafeteria for lunch, or if time is short, purchase the lunch from the hospital cafeteria. These are also the individuals who make interval telephone calls to maintain the necessary contact with the participants during follow-up. This means that we had underestimated our need for support staff by as much as 50%.

We also learned that because many of these individuals were new to research, many of the procedures that we use must be explained to them in ways that differ from the more research-experienced individuals we are more accustomed to working with. For example, the PET procedures are explained in more detail as the notion of *injecting* radioactive compounds (or any other solution) is not universally accepted without good explanation. To facilitate this process, we talk in terms of the important changes that can occur in the brain with dementia, and that we can take a picture of those changes using that injected solution.

After our participants have completed their baseline examinations, we send them a signed certificate of participation accompanied by a color image of the surface of the brain using the Freesurfer parcellations. Frequently, this results in our getting telephone calls being asked to explain “what it means.” One of the Investigators always returns these calls; it is critically important to “give back” to the communities. We also attend monthly gatherings at local Community Engagement Centers – just being present increases our familiarity to the community.

We also found that it was important to pay close attention to transportation needs. Many of our participants live in neighborhoods where public transportation is less than ideal (e.g., two or more transfers needed for a 60-min one-way trip). Consequently, we had to develop relationships with ridesharing services to obtain the quality of service that we wanted for our participants. Everyone is met at the door to the hospital by their “concierge,” and from there escorted to all of the tasks that they will do during the day. At the end of the day the ride is scheduled, and the “concierge” takes the volunteer back to the lobby and awaits the arrival of the car.

Finally, while all imaging researchers are familiar with the problem of incidental findings, the quality of those findings in a study such as this is different than that which we have encountered in the past. Many of the individuals in the study had limited healthcare resources which might have identified potential problems; many participants do not have a regular annual physical. However, we have also had instances of more severe brain injury that was a consequence of the participants living environment. One individual, for example, had suffered a severe closed head injury, and the sequalae were evident on the scan. However, there was no mention of this event despite multiple opportunities during screening and interview. The individual seemed surprised that spending more than three days in the hospital, much of the time in coma, would result in brain damage. This view is likely due in part due to lack of awareness of health-related issues.

### Comments on Preliminary Data

A significant proportion of the participants in this study have never been involved in biomedical research. Thus, our sample likely includes individuals who are typically under-represented in academic research studies and may be more representative of the population at-risk for cognitive impairment. This has resulted in the identification of a group of study participants who were cognitively impaired but had no complaints or concerns about their cognitive abilities. Further, we found that the rate of amyloid deposition among those individuals with cognitive impairment (i.e., MCI and IWOC) was lower than expected based on prior analyses (Wolk et al., 2009). Among the MCI participants 4/10 individuals (40%) recruited from the ADRC were amyloid positive, whereas only 1/16 among the individuals (6%) recruited via Pitt + Me were amyloid positive (Odds Ratio = 10.0, 95% Confidence Interval = 0.92 – 108, *p* = 0.055) [cf., (Wolk et al., 2009)].

We had assumed when the project began that participants recruited from the community would be, on average, cognitively normal; the cognitively impaired participants (and those with subjective complaints) would enroll through the ADRC. However, experience revealed a more nuanced picture. The group of individuals with impaired cognition, but who did not complain of changes in their behavior or cognition deserve special mention. The participants in this group were predominantly Black (85%) which contrasts sharply with the NC (41.7%) and SCC (14.3%) groups. Their performance on the tests used for classification was equivalent to that of the MCI participants, but without the complaints necessary for that classification. Indeed, on average the IWOC participants reported *better* cognitive abilities, and *fewer* cognitive concerns than did the cognitively normal controls. The near absence of PiB retention means that these individuals were not as yet, on the AD pathology spectrum; although with a mean age of 60 years, the amyloid cascade may not be well developed, or perhaps other non-amyloid factors may be in play [e.g., (Selkoe, 2002)].

Given the age range of the IWOC group there is also a high likelihood that these individuals (as well as other Black participants in the study) are the children or grandchildren of the people who migrated from the rural South to cities like Pittsburgh. Growing up Black in a northern city in the 1950s and 1960s was likely associated with poorer educational quality, poor access to medical care and health maintenance, as well as a range of psychosocial consequences of explicit and implicit discrimination. It may be that any racial inequities in the development of cognitive impairments are driven by pervasive institutionalized inequities that shape risk and disadvantage individuals at multiple levels, including biological, environmental, behavioral, sociocultural (Hill et al., 2015). Although these factors have often been referred to as “modifiable individual risk factors,” this term fails to recognize that individual risk is influenced by racism and social determinants *that are outside of an individual’s control*. At a population level, Black communities experience racism and more adverse social determinants of health, including negative work, living and educational conditions, that can lead to long-term negative biological consequences (Shonkoff et al., 2009; Braveman P. et al., 2011; Braveman P.A. et al., 2011). Indeed, neighborhood-level disadvantage was associated with an increased likelihood of AD neuropathology at autopsy (Powell et al., 2020). While there are established diagnostic hallmarks of AD, little attention has been paid to the possibility that factors such as neighborhood context may directly and indirectly impact brain changes that alter the connectome, thus resulting in earlier expression of the dementia. To date, little attention has been paid to the possibility that early social structural and social determinants may affect brain structure and function, alter the connectome, and reduce brain reserve and compensation resulting in the earlier expression of DAT and an apparent increased incidence of dementia among Blacks [see also Wilkins et al., 2020]. Indeed, there needs to be a paradigm shift in the field to focus on collecting the contextual and environmental data that may help disentangle apparent differences due to race; “analyzing findings by race/ethnicity without appropriate contextual data could lead to inaccurate, misleading, or stigmatizing conclusions that may detract from the overall goals of diversity in research: to enhance the accuracy, utility, and generalizability of scientific evidence” (Wilkins et al., 2020). This view is supported by the decades of research that argue that racial and socioeconomic inequities are not the result of individual behavior or biological factors but rather are due to the structures, institutional practices, and policies which contribute to adverse outcomes and susceptibilities (Fuller-Thomson et al., 2009; Nuru-Jeter et al., 2009; Mendez et al., 2014a, b; Bailey et al., 2017; Hardeman et al., 2018).

The data included in this project provides investigators around the world with the opportunity to investigate the spectrum of aging and AD effects on the brain and cognition using true-multimodal imaging, and detailed cognitive/behavioral evaluations. Genetic analyses will be completed starting at the end of 2020, and those restricted data will be available directly from the study investigators. Longitudinal follow-up of the individuals in the study is underway, and there are plans to enrich the sample of pre-DAT participants and continue follow-up. These data, combined with the main HCP dataset, the HCP Lifespan and Aging datasets, and the other CRHD project related to AD provide richest and most comprehensive resource for the neurobiological study of AD and related dementias.

## Conclusion

The study has two unique characteristics. First, the data are acquired using standard and standardized procedures that are shared by other CRHD studies, including the HCP Lifespan Study (Bookheimer et al., 2019). This provides an international, accessible database for all investigators. Second, and more important, are the characteristics of the study sample. We used multiple portals of entry, including customized web sites that allowed to achieve our goal of ∼50% Black participants, and reaching people who were participating in their first research study. This, we believe, at least partly explains why our measured rates of AD pathology are lower than those in more typical research samples [e.g., (Wolk et al., 2009)]. In addition, we identified a group of participants whose test performance was as poor as that of the MCI participants, but who reports few concerns about their cognition [c.f., (Antinori et al., 2007)]; this group is predominately Black.

This leads us to what we believe is the most important implication of our data, and which is a weakness of the study as currently described. Specifically, we, like many others, make the mistake of “analyzing [our] findings by race/ethnicity without appropriate contextual data [which] could lead to inaccurate, misleading, or stigmatizing conclusions that may detract from the overall goals of diversity in research: to enhance the accuracy, utility, and generalizability of scientific evidence” (Wilkins et al., 2020). Race is a socially determined construct that is not biologically or genetically based (Cooper and David, 1986). In addition to strong data suggesting there are no biologically determined differences between races (Serre and Paabo, 2004), defining race as a social construct has the advantage of capturing the concept of racism more precisely (Jones, 2000). Racism is thus better defined as a system that structures opportunity based on race, providing unfair advantages and disadvantages based on race.

There is still considerable disagreement on the factors contributing to disparities in many AD- related outcomes, e.g., dementia onset and course. Much of this is likely due to the focus on individual behavior or “lifestyle factors” without consideration for the social, physical, and policy environments that are inextricably linked to the individual and are key to understanding health disparities (Cooper et al., 2015). Perhaps a better way to place the factors related to AD and dementia into the NIA Health Disparities Framework is to study the interplay between social determinants of health, racism, and AD and dementia (Hill et al., 2015). Aside from the more direct effects racism on risk factors, we also believe that racism may have the moderating effect of *reducing* the impact of the positive social determinants of health (SDOH) (e.g., education, access to health care) and *increasing* the impact of negative SDOH (e.g., poverty, social isolation). Significant advances in AD and dementia prevention and management will be made as we accumulate more information SDOH and how racism affects their relationship with resilience, diagnosis, prognosis, and response to treatment.

## The CoBRA Study Team

The CoBRA Study team includes: Ann D. Cohen, Beth E. Snitz, Ted Huppert, Tae Kim, Yue-Fang Chang, Yu Cheng, Jack Doman, Ricardo Bruña, Fernando Maestu, Avniel Ghuman, Anto Bagic, Ilyas Kamboh, Anne Newman, Steven Reis, Rebecca Roush, Lara Fatukasi, Leslie Kovach, Katey Potopenko, Howard J. Aizenstein, Lewis H. Kuller, William E. Klunk, Caterina Rosano, Oscar L. Lopez, and James T. Becker.

With the exceptions of AC and JB, the order of the authors is alphabetical.

## Data Availability Statement

The datasets presented in this study can be found in online repositories. The names of the repository/repositories and accession number(s) can be found below: HCP Connectome Central Facility https://www.humanconnectome.org/study/connectomics-brain-aging-and-dementia; NIMH Data Archive https://nda.nih.gov/edit_collection.html?id=3159.

## Ethics Statement

The studies involving human participants were reviewed and approved by Human Research Protection Office; University of Pittsburgh. The patients/participants provided their written informed consent to participate in this study.

## Author Contributions

AC and JB made the initial draft of the work while the remaining authors revised it for important intellectual content. All authors made substantial contributions to the conception and design of the work, as well as the acquisition, analysis, interpretation of data for the work, and had final approval of the version to be published.

## Conflict of Interest

The authors declare that the research was conducted in the absence of any commercial or financial relationships that could be construed as a potential conflict of interest.

## Publisher’s Note

All claims expressed in this article are solely those of the authors and do not necessarily represent those of their affiliated organizations, or those of the publisher, the editors and the reviewers. Any product that may be evaluated in this article, or claim that may be made by its manufacturer, is not guaranteed or endorsed by the publisher.

## Footnotes

1
https://grants.nih.gov/grants/guide/pa-files/par-14-281.html

2
https://en.wikipedia.org/wiki/Human_Connectome_Project

3
https://www.humanconnectome.org/

4
https://nda.nih.gov/

5
https://www.adrc.pitt.edu/

6
https://pittplusme.org/studyarms/publicdetails?guid=abdb4de3-0e00-49e6-b0a0-a3ca2d6e7c2a

7
http://www.neurology.upmc.edu/cabmsi/

8
http://www.healthmeasures.net/explore-measurement-systems/nih-toolbox

9
http://www.healthmeasures.net/explore-measurement-systems/promis

10
https://www.xnat.org/

11
http://www.neuro.uni-jena.de/cat/

12
http://www.pmod.com

13
http://surfer.nmr.mgh.harvard.edu

14
http://www.healthmeasures.net/index.php?option=com_instruments&view=measure&id=847&Itemid=992

15
https://www.humanconnectome.org/study/connectomics-brain-aging-and-dementia
