# Supplementary material for: Connectomics in Brain Aging and Dementia – The Background and Design of a Study of a Connectome Related to Human Disease
Source: Front Aging Neurosci. 2021 Oct 7;13:669490. doi: 10.3389/fnagi.2021.669490 (PMC8530182; doi:10.3389/fnagi.2021.669490)
Supplement: Supplementary file 1 [file Data_Sheet_1.docx]

| **Supplemental Table 1: Sensory/Motor/Cognitive/Emotional Measures** | |
| --- | --- |
| **Domain** | **Subdomain (Measure Name)** |
| **Alertness** | Montreal Cognitive Assessment |
|  | Sleep (Pittsburgh Sleep Questionnaire) |
| **Cognition** | Episodic Memory (Picture Sequence Memory) |
|  | Executive Function/Cognitive Flexibility  (Dimensional Change Card Sort) |
|  | Executive Function/Inhibition (Flanker Inhibitory Control and Attention Task) |
|  | Language/Reading Decoding  (Oral Reading Recognition) |
|  | Language/Vocabulary Comprehension  (Picture Vocabulary) |
|  | Processing Speed  (Pattern Comparison Processing Speed) |
|  | Working Memory (List Sorting) |
| **Emotion** | Negative Affect (Sadness, Fear, Anger) (self-report) |
|  | Psychological Well-being (Positive Affect, Life Satisfaction, Meaning and Purpose) (self-report) |
|  | Stress and Self-Efficacy  (Perceived Stress, Self-Efficacy) (self-report) |
| **Motor** | Endurance (2 minutes walk test) |
|  | Locomotion (4-meter walk test) |
|  | Dexterity (9-hole Pegboard) |
|  | Strength (Grip Strength Dynamometry) |
| **Sensory** | Audition (Words in Noise) |
|  | Olfaction (Odor Identification Test) |

| **Supplemental Table 2: Personal Factors and Psychiatric Assessment** | |
| --- | --- |
| **Domain** | **Subdomain (Measure Name)** |
| **Personality** | Five Factor Model (NEO-FFI) |
| **Health and Family History** | Height, Weight, BMI |
|  | Hematocrit and fasting HgA1c |
|  | Fasting Glucose and Insulin |
|  | Menstrual Cycle Information |
| **Psychiatric and Life Function** | Life Function (Achenbach Adult Self-Report, Syndrome Scales and DSM-Oriented Scale) |
|  | Parental History of Psychiatric or Neurologic illnesses |
| **Substance Abuse** | Alcohol, Smoking and Substance Involvement Screening Test (ASSIST) |

| **Supplemental Table 3: Measures Related to Risk for and Protection from Cognitive Impairment** | |
| --- | --- |
| **Domain** | **Measure** |
| **Exercise/Motor Function** |  |
|  | Actigraphy |
|  | Blocks walked per week |
|  | Kcal/week |
|  | Gait speed |
| **Cognitive Stimulation** |  |
|  | Florida Cognitive Activities Scale |
| **Body Habitus** |  |
|  | Waist-Hip Ratio |
|  | Nutrition/Diet |
| **Vascular Health** |  |
|  | Standardized Blood Pressure |
| **Inflammation** |  |
|  | C-Reactive Protein |
|  | Interleukin-6 |
|  | Tumor Necrosis Factor-α |
| **Metabolic/Pulmonary** |  |
|  | Fasting Lipid Profile |
|  | Homocysteine |
|  | Cystatin-C, eGFR |

| **Day 1** | **Name** | **Acq Time** | **TR (ms)** | **TE (ms)** | **Flip°** | **Voxel size (mm)^3^** | **FOV (mm)** | **Bandwidth (Hz/Px)** | **Echo spacing (ms)** | **.edate file name** |
| --- | --- | --- | --- | --- | --- | --- | --- | --- | --- | --- |
| **Localizer** |  |  |  |  |  |  |  |  |  | NA |
| **AAHead_Scout_64ch_head-coil** |  |  |  |  |  |  |  |  |  | NA |
| **Localizer_align** |  |  |  |  |  |  |  |  |  | NA |
| **Structural** | T1W_MPR | 6:38 | 2400 | 2.22 | 8 | 0.8 | 256 | 220 | 7.5 | NA |
|  | T2W_SPC | 5:57 | 3200 | 563 | 8 | 0.8 | 256 | 744 | 3.52 | NA |
|  | T2W_FLAIR | 5:21 | 9690 | 91 | 150 | 0.8 | 256 | 223 | 9.09 | NA |
|  | T2W_SWI | 3:01 | 28 | 20 | 15 | 0.8 | 256 | 120 |  | NA |
| **Resting State fMRI** | BOLD_REST1_AP | 5:46 | 800 | 37 | 52 | 2.0 | 208 | 2290 | 0.58 | NA |
| **Resting State fMRI** | BOLD_REST2_PA | 5:46 | 800 | 37 | 52 | 2.0 | 208 | 2290 | 0.58 | NA |
| **Bias_BC** |  |  |  |  |  |  |  |  |  | NA |
| **Bias_BC_64channel** |  |  |  |  |  |  |  |  |  | NA |
| **Fieldmap** | GRE | 2:15 | 731 | 4.92 | 7.38 | 2 | 208 | 566 |  | NA |
| **SE - Fieldmap AP** | SE-EPI | 0:32 | 8000 | 66 |  | 2 | 208 | 2290 |  | NA |
| **SE - Fieldmap PA** | SE-EPI | 0:32 | 8000 | 66 |  | 2 | 208 | 2290 |  | NA |
| **Resting State fMRI** | BOLD_REST2_AP | 5:46 | 800 | 37 | 52 | 2.0 | 208 | 2290 | 0.58 | NA |
| **Resting State fMRI** | BOLD_REST2_PA | 5:46 | 800 | 37 | 52 | 2.0 | 208 | 2290 | 0.58 | NA |
| **Task fMRI** | BOLD_WM1_AP | 5:07 | 800 | 37 | 52 | 2.0 | 208 | 2290 | 0.58 |  |
| **Task fMRI** | BOLD_WM2_PA | 5:07 | 800 | 37 | 52 | 2.0 | 208 | 2290 | 0.58 |  |
| **Rest ASL** | ASL | 6:13 | 4600 | 16.18 | 180 | 1.5 | 192 | 2694 | 0.5 | NA |

**Supplemental Table 4: HCP MRI Scan Protocol**

| **Day 2** | **Name** | **Acq Time** | **TR (ms)** | **TE (ms)** | **Flip°** | **Voxel size (mm)^3^** | **FOV (mm)** | **Bandwidth (Hz/Px)** | **Echo spacing (ms)** | **. edate file name** |
| --- | --- | --- | --- | --- | --- | --- | --- | --- | --- | --- |
| **Localizer** |  |  |  |  |  |  |  |  |  | NA |
| **AAHead_Scout_64ch_head-coil** |  |  |  |  |  |  |  |  |  | NA |
| **Localizer_align** |  |  |  |  |  |  |  |  |  | NA |
| **Resting State fMRI** | BOLD_REST3_AP | 5:46 | 800 | 37 | 52 | 2.0 | 208 | 2290 | 0.58 | NA |
| **Resting State fMRI** | BOLD_REST3_PA | 5:46 | 800 | 37 | 52 | 2.0 | 208 | 2290 | 0.58 | NA |
| **Resting State fMRI** | BOLD_REST4_AP | 5:46 | 800 | 37 | 52 | 2.0 | 208 | 2290 | 0.58 | NA |
| **Resting State fMRI** | BOLD_REST4_PA | 5:46 | 800 | 37 | 52 | 2.0 | 208 | 2290 | 0.58 | NA |
| **Task fMRI** | BOLD_MOTOR1_AP | 3:38 | 800 | 37 | 52 | 2.0 | 208 | 2290 | 0.58 |  |
| **Task fMRI** | BOLD_MOTOR2_PA | 3:38 | 800 | 37 | 52 | 2.0 | 208 | 2290 | 0.58 |  |
| **Bias_BC** |  |  |  |  |  |  |  |  |  | NA |
| **Bias_BC_64channel** |  |  |  |  |  |  |  |  |  | NA |
| **Fieldmap** | GRE | 2:15 | 731 | 4.92 | 7.38 | 2 | 208 | 566 |  | NA |
| **SE - Fieldmap AP** | SE-EPI | 0:32 | 8000 | 66 |  | 2 | 208 | 2290 |  | NA |
| **SE - Fieldmap PA** | SE-EPI | 0:32 | 8000 | 66 |  | 2 | 208 | 2290 |  | NA |
| **Diffusion weighted** | dMRI_dir98_AP | 5:37 | 3230 | 89.2 | 78 | 1.5 | 210 | 1700 | 0.69 | NA |
| **B-value 0, 3000** | dMRI_dir98_PA | 5:37 | 3230 | 89.2 | 78 | 1.5 | 210 | 1700 | 0.69 | NA |
| **Phase 6/8** | dMRI_dir99_AP | 5:41 | 3230 | 89.2 | 78 | 1.5 | 210 | 1700 | 0.69 | NA |
|  | dMRI_dir99_PA | 5:41 | 3230 | 89.2 | 78 | 1.5 | 210 | 1700 | 0.69 | NA |
|  |  |  |  |  |  |  |  |  |  |  |
|  |  |  |  |  |  |  |  |  |  |  |

| **Supplemental Table 5: List of SNPs to be examined in genetic analyses** | | |
| --- | --- | --- |
| **SNP** | **Closest Gene** | **Proposed Function** |
| rs6656401 | CR1 | regulation of the complement cascade and clearance of immune complexes |
| rs6733839 | BIN1 | form complexes that act in clathrin-mediated endocytosis |
| rs10948363 | CD2AP | cytoskeletal organization and required for synapse formation. |
| rs11771145 | EPHA1 | synapse formation and in plasticity |
| rs9331896 | CLU | binding lipids and Aβ |
| rs983392 | MS4A6A | immune respone |
| rs10792832 | PICALM | clathrin-mediated endocytosis |
| rs4147929 | ABCA7 | lipid metabolism |
| rs3865444 | CD33 | immune response |
| rs9271192 | HLA-DRB5;  HLA-DRB1 | immune response |
| rs28834970 | PTK2B | neuronal calcium influx and signaling |
| rs11218343 | SORL1 | APP processing and generation of Aβ |
| rs10498633 | SLC24A4-RIN3 | neuronal development and hypertension risk |
| rs8093731 | DSG2 | encodes cadherin proteins |
| rs35349669 | INPP5D | immune response |
| rs190982 | MEF2C | immune response and synapse function |
| rs2718058 | NME8 | cytoskeletal function and axonal transport |
| rs1476679 | ZCWPW1 | modulation of epigenetic regulation |
| rs10838725 | CELF1 | cytoskeletal function and axonal transport |
| rs17125944 | FERMT2 | Tau metabolism |
| rs7274581 | CASS4 | APP and Tau metabolism and in cytoskeletal function and axonal transport |
