# Supplementary figures and images for: Connectomics in Brain Aging and Dementia – The Background and Design of a Study of a Connectome Related to Human Disease
Source: Front Aging Neurosci. 2021 Oct 7;13:669490. doi: 10.3389/fnagi.2021.669490 (PMC8530182; doi:10.3389/fnagi.2021.669490)

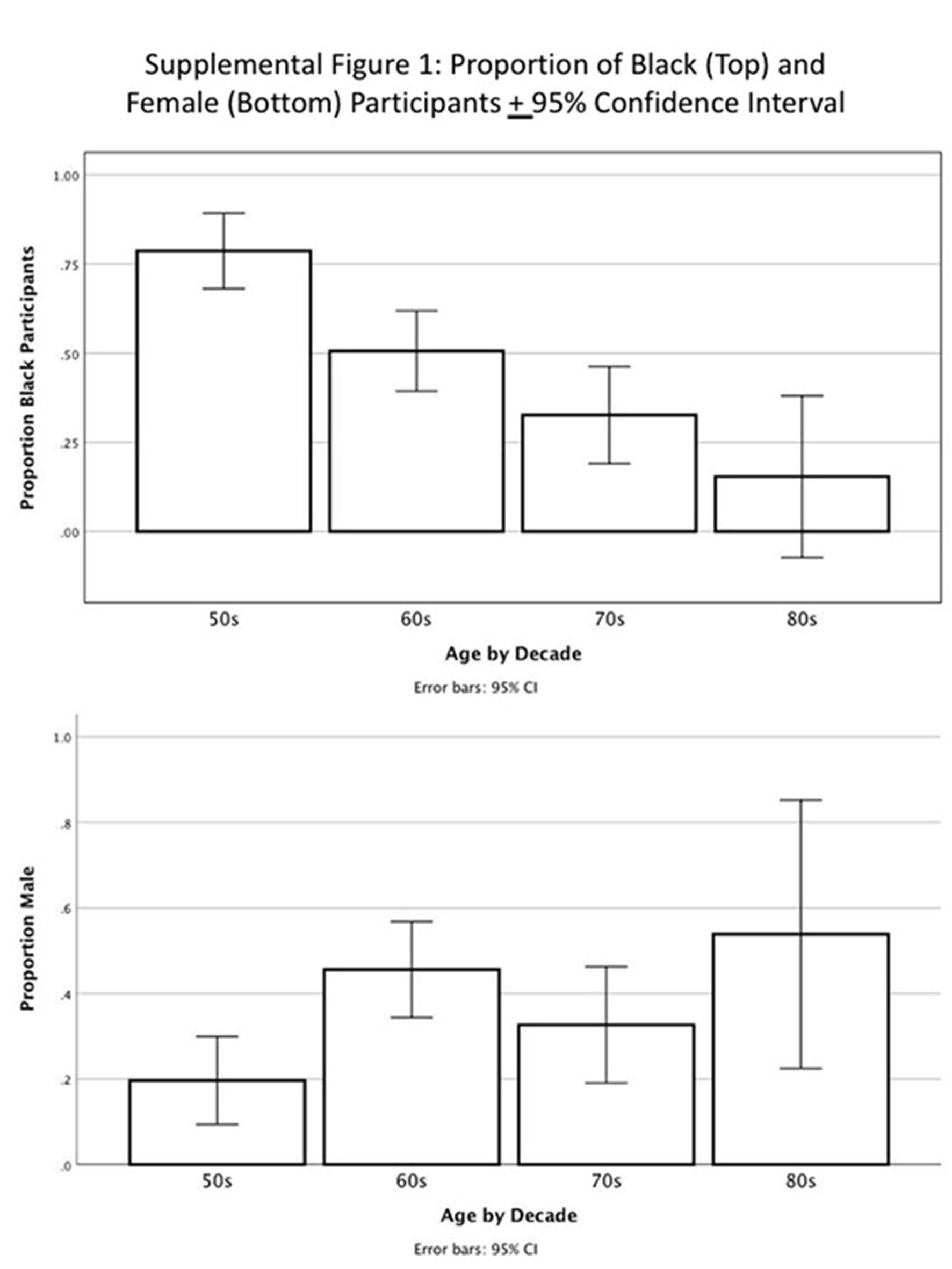

Supplement: Supplementary file 2 [file Image_1.JPEG]
